# Molecular and Circulating Biomarkers of Brain Tumors

**DOI:** 10.3390/ijms22137039

**Published:** 2021-06-29

**Authors:** Wojciech Jelski, Barbara Mroczko

**Affiliations:** 1 Department of Biochemical Diagnostics, Medical University, 15-269 Bialystok, Poland; mroczko@umb.edu.pl; 2 Department of Neurodegeneration Diagnostics, Medical University, 15-269 Bialystok, Poland

**Keywords:** tumor markers, brain tumor

## Abstract

Brain tumors are the most common malignant primary intracranial tumors of the central nervous system. They are often recognized too late for successful therapy. Minimally invasive methods are needed to establish a diagnosis or monitor the response to treatment of CNS tumors. Brain tumors release molecular information into the circulation. Liquid biopsies collect and analyze tumor components in body fluids, and there is an increasing interest in the investigation of liquid biopsies as a substitute for tumor tissue. Tumor-derived biomarkers include nucleic acids, proteins, and tumor-derived extracellular vesicles that accumulate in blood or cerebrospinal fluid. In recent years, circulating tumor cells have also been identified in the blood of glioblastoma patients. In this review of the literature, the authors highlight the significance, regulation, and prevalence of molecular biomarkers such as O_6_-methylguanine-DNA methyltransferase, epidermal growth factor receptor, and isocitrate dehydrogenase. Herein, we critically review the available literature on plasma circulating tumor cells (CTCs), cell-free tumors (ctDNAs), circulating cell-free microRNAs (cfmiRNAs), and circulating extracellular vesicles (EVs) for the diagnosis and monitoring of brain tumor. Currently available markers have significant limitations. While much research has been conductedon these markers, there is still a significant amount that we do not yet understand, which may account for some conflicting reports in the literature.

## 1. Introduction

Central nervous system (CNS) tumor is one of the most malignant tumors in humans. Itaccounts for approximately 1.35% of all malignant neoplasms and 2.95% of cancer-related deaths [1]. The pathogenesis of brain tumors is still poorly understood. Genetic predisposition plays an important role. In addition, risk factors include various environmental factors such as low-frequency electromagnetic fields, chemical agents, head trauma, tobacco use, alcohol, and infections. It is likely that the interaction between genetic and environmental factors, known as a gene–environment interaction, may also increase the risk of CNS tumors [2]. Brain tumors are a diverse group of neoplasms with different types of primary brain tumors or metastatic cancer. The most common malignant brain tumors are glioblastomas that originate from glial cells. They are avery aggressive histological type of cranial tumor with a 5-year survival rate of less than 5% [1]. Brain tumors are usually diagnosed after clinical signs include headache, dizziness, vomiting, personality changes, or focal neurological disorders. Currently, magnetic resonance imaging (MR) is the main imaging method in patients suspected of having a brain tumor. For the final diagnosis of tumor type and grade of malignancy, examination of the tumor tissue obtained by biopsy or resection is required [3]. Unfortunately, once a tumor is detected under the microscope, it is often too late for effective treatment. The prognosis in patients is correlated with the stage of the disease at the time of detection, and therefore, it is important to find markers that allow the early detection of the tumor. The development of a tumor is a complex process leading to a number of biochemical and molecular changes. Diagnostic tests to detect these changes using biomarkersshow significant potential for early detection. In the last few years, several diagnostic and prognostic biomarkers of malignant glioblastomas have been described [4]. These markers significantly contributed to the accuracy of diagnosis and the effectiveness of therapy. Minimally invasive diagnostic methods are valuable in a clinical approach to establishing the diagnosis and monitoring treatment for brain tumors. Liquid biopsy by cerebrospinal fluid or blood sampling holds promise in this regard. As a result of the liquid biopsy method, the tumor components in the body fluids are collected and analyzed. The liquid biopsy method is gaining more and more interest as a substitute for neoplastic tissue [5]. In this review, we provide an overview of molecularblood biomarkers for brain tumor detection.

**Figure 1:**
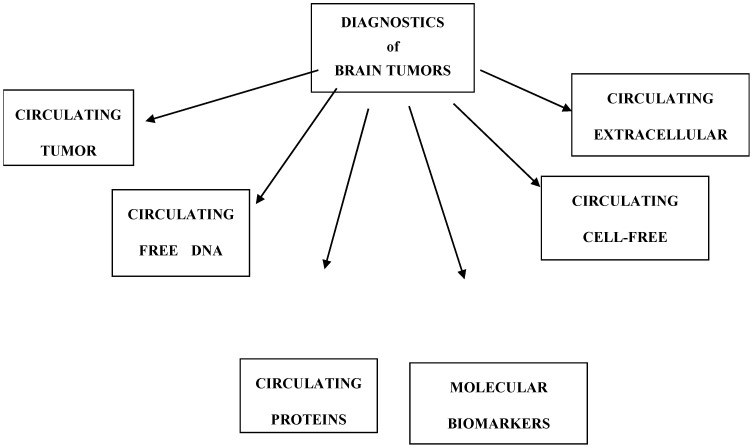
Division of brain tumor markers.

Tumor markers can be categorized into several groups: molecular biomarkers, circulating tumor cells (CTCs), circulating free DNA (cfDNA), circulating cell-free microRNAs (cfmiRNAs), and circulating extracellular vesicles (EVs) (Figure 1, Table 1) [6].

## 2. Molecular Biomarkers

Although molecular markers are widely used to differentiate particular types of cancer, very few provide reliable and reproducible predictive markers. Due to editorial limitations, we only discuss some of the more well-known molecular markers of brain tumors.

The gene encoding O-6 methylguanine-DNA methyltransferase (MGMT) is located on the chromosome at position 10q26. O-6 methylguanine-DNA methyltransferase is involved in DNA repair by reversing DNA alkylation and removing the guanine-alkyl group, which prevents apoptosis [28]. Expression of this protein is strongly modulated by different transcription factors such as specificity protein 1 or nuclear factor kappa B, which activate O-6 methylguanine-DNA methyltransferase promoter to induce the expression of more MGMT [7]. Promoter methylation of MGMT occurs in approximately 40% of glioblastoma. MGMT promoter methylation is found more frequently (approximately 75%) in secondary glioblastoma because it correlates strongly with the TP53 mutation (92%) in secondary GBM and is only in 36% of primary tumors [29]. O-6 methylguanine-DNA methyltransferase expression is associated with DNA-resistant alkylating agents such as temozolomide, the major chemotherapeutic agent used in glioblastoma. Low levels of this protein correlate with slightly longer survival and response to temozolomide. Hegi and coworkers showed that MGMT promoter methylation gives better results in patients receiving TMZ. Median overall survival in cases with current methylation was 18.2 months and 12.2 months in cases without methylation [30]. Becausetemozolomide is toxic, detecting the methylation status of the MGMT promoter can help determine the best TMZ therapy.

The most common method of detecting methylation MGMT in glioblastoma is a polymerase chain reaction (PCR) method or combinatorial PCR with MS technology, SYBR Green, or pyrosequencing in glioblastoma patients [8].

The epidermal growth factor receptor (EGFR) is a major activator of various signaling pathways and physiological responses, including migration, proliferation, survival, and tumor formation. EGFR is a potential glioblastoma biomarker. In healthy cells, it is involved in growth factor signaling, while cancer-related oncogenic changes (variant expression, mutations) often confer ligand-independent oncogenic activity. EGFR is amplified in approximately 40% of glioblastoma patients and is often associated with high-grade tumors. There can be several dozen additional copies of EGFR in tumors [9]. EGFR is encoded by a gene of the same name that encodes a tyrosine kinase receptor specific for certain growth factors. In a brain tumor, one of the most commonly studied changes in EGFR is EGFR transcript variant III (EGFRvIII), This mutation is due to a histone modification on its enhancer gene on chromosome 7p12 [10]. Overexpression of EGFRvIII in the presence of epidermal growth factor receptor amplification has been proposed as the strongest indicator of poor prognosis and survival [10]. However, other researchers suggest that EGFRvIII may be a positive prognostic marker and indicate the long-term survival of EGFRvIII patients treated with surgery/chemotherapy/radiotherapy [31,32]. This molecular marker may also be considered a predictor of response to receptor tyrosine kinase (RTK) inhibitors. Although EGFR-amplified tumors initially respond to RTK inhibition, data suggest that they are often refractory to this treatment [28]. EGFR mutation and amplification were classified as prognostic biomarkers because they are abundant in glioblastoma cells. Due to the proliferative nature of the tumor, controlled mainly by key growth factors and their receptors, EGFR can activate pathways necessary for the development of GBM cancer cells [33].

Isocitrate dehydrogenase (IDH) is a protein enzyme encoded by genes on chromosome 2, whose main function is to catalyze oxidative decarboxylation in the Krebs cycle. IDH has been grouped into two classes (IDH 1 and IDH 2). These isoenzymes catalyze the reversible oxidation of isocitrate to form α-ketoglutarate while reducing NADP + to NADPH. NADPH provides cell-free protection against intracellular oxidative injury [34]. The most common IDH 1 or 2 mutation is a single-residue change that replaces histidine instead of arginine, thereby converting alpha-ketoglutarate (a-KG), a normal product, to D-2-hydroxy-glutarate (D-2HG), which is an oncometabolite. How this promotes tumorigenesis is currently unknown, but it is probably related to the effects of D-2-hydroxy-glutarate on DNA demethylases, which promotes DNA and histone methylation. D-2HG has also been used as a biomarker of response to treatment [35]. Mutations in isocitrate dehydrogenase are found in 73–85% of secondary GBM, whereas it is rarely present in primary GBM [11]. Tao et al. showed that IDH1-mutant cells are more sensitive to radiotherapy than wild-type cells and gliomas with a secondary IDH1 mutation show increased chemosensitivity [36]. Therefore, IDH mutations are considered a positive prognostic marker of survival in stage II to IV glioblastomas [12]. Detection of this biomarker is possible using immunohistochemistry or spectroscopy. The main limitation of the use of this biomarker is the determination of the fate of the IDH mutation in the progression of a tumor. Isocitrate dehydrogenase mutations can also be detected by techniques such as pyrosequencing or droplet-type digital polymerase chain reaction (ddPCR) [37].

Glial fibrillary acidic protein (GFAP) is an intermediate fiber protein produced by astrocytes and other cells of the central nervous system. It is detected at a much higher level in tumor tissue compared to normal brain cells [13]. However, GFAP in the blood cannot be used as a specific diagnostic marker for a brain tumor because it exhibits a so-called "sensitivity gap" due to the heterogeneous/low expression of GFAP on some tumors, which leads to undetectable amounts of GFAP released into the bloodstream. Elevated concentrations of GFAP in the blood correlate with tumor volume, intratumor GFAP expression, and the degree of necrosis [14]. Moreover, serum GFAP concentration is associated with isocitrate dehydrogenase mutational status [38]. Glial fibrillary acidic protein is presently the most prevalent marker for the identification of circulating tumor cells, and expression is frequently maintained in brain tumors, despite its heterogeneity.

Telomerase reverse transcriptase (TERT) is an enzyme of the ribonucleoproteinase family involved in replication telomeres at the end of the chromosomes, which contain repetitive DNA sequences. They gradually shorten during successive cell divisions, ultimately leading to a permanent proliferative arrest. Telomerase is the primary enzyme that prevents telomere shortening through cell division and only expressed in stem cells. It is also crucial for cell transformation and immortalization during cancer development [15]. Two specific point mutations in the promoter of telomerase (pTERT), C228T and C250T, have been reported in cancer cells and are proposed to activate telomerase [15]. pTERT mutations have been found in a variety of cancers, including brain tumors, although they have not been found in normal cells. A high percentage of brain tumor samples have pTERT mutations correlated with increased levels of the TERT gene protein [16]. Due to the high frequency of occurrence, pTERT mutations detected in liquid biopsy may be a prognostic factor for brain tumors, and detection could contribute to future diagnostic tests [39].

Loss of heterozygosity (LOH) is the loss of genetic material from one of the two alleles of certain genes. LOH is common in malignant neoplastic cells, which mainly affects tumor suppressor genes and then leads to reduced protection of the body’s systems against tumor formation [17]. It is a common genetic event in brain tumors. LOH 10q is present in 60–80% of primary and secondary brain tumors. The three commonly deleted loci on chromosome 10 are 10q23-24 (PTEN), 10q14-p15, and 10q25-pter. The most important of these three is the loss of the tumor suppressor gene PTEN along with genes including MXI1, DMBT1, LGI1, FGFR2, and WDRI1. The PTEN protein is a phosphatase that plays an important role in inhibiting the PI3K/AKT/mTOR pathway. PTEN mutations or loss of favored tumor development and loss of the PTEN loci 10q25-pter is associated with the progression of low-grade brain tumors to high-grade glioblastomas [18]. LOH on chromosome 22 has also been found in brain tumors. The most common loss is chromosome 22q, which is present in 41% of the primary tumor and 82% of the secondary tumor. Deletion of the 22q12.3 locus leads to the loss of the tumor suppressor gene TIMP-3, which encodes the tissue inhibitor of metalloproteinases-3 (TIMP-3). TIMP-3 inhibits tumor cell growth and cancer progression and induces apoptosis [40]. Within brain tumors, many chromosomes are affected with LOH, namely 1p, 9p, 17p, and 19q [41]. Chromosome 19q LOH is more often detected in the secondary brain tumor than in the primary one. LOH on chromosome 1 is a rare genetic event in primary (12%) and secondary brain tumors (15%) but is associated with longer survival [42]. LOH analysis in brain tumor patients is performed using microsatellites and PCR to amplify gene products.

The TP53 gene encodes the well-known tumor suppressor protein p53. It is considered the guardian of the genome, and p53 has various roles in inhibiting tumor formation. TP53 point mutations were found much more often in secondary (90%) compared to primary brain tumors (30%), and in some cases, they were not found at all in primary lesions [19]. One of the proposed mechanisms of TP53 mutation in supporting the progression of brain tumors is via the mevalonate (MVA) pathway regulation. Based on qRT-PCR methods, it was verified that the TP53 mutation is upregulated and correlated with activation of the MVA pathway due to upregulation of enzymes known to promote tumor formation, namely MVA kinases and 3’-hydroxy-3’-methylglutaryl-coenzyme A reductase [20]. The use of inhibitors of mouse double minute 2 homolog is efficacious in patients harboring TP53 mutations [23].

## 3. Circulating Tumor Cells

Circulating tumor cells (CTCs) are cells shed from primary or metastatic tumors into the body fluids, including blond, cerebrospinal fluid, and urine. CTC determines the ability of epithelial tumor cells to metastasize. It has not been decided whether CTCs are just central tumor subpopulations or rather randomly selected representing the entire primary tumor. CTCs have been shown to play an important role in the development of metastasis; however, the processes by which CTCs leave the tumor, enter the bloodstream, and attack target organs for metastatic colonization are very complex and have not been fully elucidated [43]. The results of many studies have shown that CTCs are an effective biomarker for predicting the prognosis in various cancers, e.g., melanoma, lung cancer, and pheochromocytoma [44]. Additionally, in patients with glioblastoma, circulating tumor cells are similarly detected, which can lead to disease spread and metastasis. CTCs can also serve in themonitoring of glioblastoma patients. Interestingly, their levels detected after chemotherapy are significantly lower compared to the levels before treatment, which may provide invaluable insight in differentiating tumor progression from radiation necrosis [21]. The importance of CTC in CNS tumors has been fully confirmed in many studies. CTC provides good and minimally invasive samples for tumor detection. Specific CTC genotypes may reflect the progression of the primary tumor and the change in specific genetic information during the relapse process. Circulating tumor cells have been reported to be prevalent in glioblastoma, up to about 75% [45]. It is worth noting that CTCscan be a surrogateof tumor tissue andanalyzed for the presence of molecular biomarkers [46]. Although CTCs show great potential for their application in the diagnosis of glioblastoma, their implementation into the clinical environment is associated with many challenges. Detection of CTCs from GBM patients seems to be limited by low CTC flow and method complexity [21]. The most commonly used CTC monitoringmethods rely on the presence the epithelial cell adhesion molecules (EpCAMs), which are expressed on the surface of most cancer cells but not glioblastoma cells. Therefore, other strategies have been employed to detect GBM CTCs. Easily accessible from body fluids such as blood samples and analyzed by telomerase tests or EGFR amplification [22]. The frequency of CTC release into the peripheral blood from brain tumors has not yet been finalized and may not be a ubiquitous biomarker with treatment.

## 4. Circulating Free DNA

Circulating tumor DNA (ctDNA)are DNA fragments released into the bloodstream by the breakdown of cancer tissue. In patients without cancer, blood cfDNA comes mainly from genomic DNA released during the inflammation process or cell apoptosis [47]. Genomic cfDNAs are long DNA fragments (>500 bp). In physiological conditions, the concentration of cfDNA in the blood is low due to its removal by phagocytes. In advanced solid tumors, higher levels of ctDNA are found in the blood, and this has been extensively studied. Several studies have shown that ctDNA have been detected in some patients with primary CNS tumors, including astrocytoma and oligoastrocytoma. Glioblastoma differs from other neoplasms in that the concentration and positive index of ctDNA in the serum of patients are low [24].

Once extracted, detection of somatic alterations on ctDNA depends on the quantity of ctDNA and the sensitivity of the sequencing method. The ctDNA proportion among whole cfDNA is correlated with tumor burden in advanced solid tumors. The ctDNA dynamically reflects tumor progression and provides the specific mechanism of primary tumor gene mutation and drug resistance. In addition, ctDNAs show the molecular composition of tumors in patients with CNS tumors, including data on targeted mutations and drug resistance mechanisms in selective therapy. CtDNA analysis can detect tumor progression and drug resistance mutations at an early stage. This information can provide more effective data at an early stage and improve treatment effectiveness [24].

As themean half-life duration of ctDNA is short (~1.5–2 h), plasma must be quickly separated and frozen within 3 h aftercollection [25]. Bettegowda et al. found that CSF is better than serum as a sample for the detection of ctDNAs derived from primary brain tumors [48]. The research of Wang et al. indicated that 74% of the samples contained primary tumor DNA, and the degree of tumor DNA detection was related to the anatomical location and grade of the tumor, but not to its size [49].

## 5. Circulating Free miRNA

MicroRNA (miRNA) areshort noncoding RNA molecules (<25 pz) that complete the post-transcriptional level regulation of gene expression by degrading or inhibiting target mRNA [50]. MicroRNAs play key roles in the homeostasis and intercellular communication of both healthy and malignant tissues [51]. The role of miRNA in the development and progression of tumor cells is based on modulating growth, apoptosis, and differentiation processes, especially in glioblastoma [52]. Analysis of miRNA accurately identified cancer tissue origin in a variety of cancers. In recent years, many studies have analyzed serum miRNA signature in human glioblastoma as diagnostic or prognostic markers. Dong et al. identified 24 miRNAs that were significantly reduced and 115 miRNAs that were significantly elevated in the serum of GBM patients, but not healthy controls [53]. For example, miR-21 is an important miRNA studied in cancer, and itsupregulation has been reported in the plasma and tissueof glioblastoma patients and associated with lower overall survival and tumor grading [54]. It is an anti-apoptotic factor in glioblastoma cell lines thatacts through caspase inhibition. The inhibition of miR-21 subsequently halts cell growth, increases apoptosis, and reduces proliferation of GBM cancer. It is also hypothesized to have a role in cancer stem cell (CSC) differentiation due to its upregulation in the glioblastoma CSC population via the Fas ligand as its main genetic target. MiR-21 in glioblastoma has been confirmed as a specific tumor markerfor predicting overall survival and treatment response. MiR-21 is highly expressed in other various types of cancers (i.e., lung, cervix, ovaries) [55]. Tang et al. found significantly higher circulating levels of miR-185 in glioblastoma patients compared to healthy controls. Interestingly, the levels of this miRNA returned to normal levels after surgery and chemoradiotherapy [26]. On the other hand, several studies have shown that low serum levels of miRNA-125b seem to be associated with the diagnosis of glioblastoma [27]. Additionally, Wang et al. described three miRNAs, miR-128, miR-485-3p, and miR-342-3p, which are downregulated in patients when compared with healthy controls. Their levels correlated with glioblastoma grades and increased after surgery and chemoradiation, suggesting their use as biomarkers toassess tumor grading and to monitor treatment response [56].

MiRNAs are stable in body fluids and could be detected by PCR.miRNA samples are often taken from body fluids (e.g., blood and CSF) for glioblastoma profiling. The use of miRNAs as biomarkers gives >90% specificity in the detection of glioblastoma. MiRNA is a useful biomarker for cancer detection due to its minimally invasive approach, obtained mainly from body fluids, and also allows for the stratification of patients in terms of the predictive and prognostic capacity of molecular biomarkers [57].

## 6. Circulating Extracellular Vesicles

Extracellular vesicles (EVs) are small (generally 40–100 nm in diameter) lipid-bilayer-enclosed vesicles released by both cancer and noncancer cells into the extracellular space. They carry various cell componentssuch as proteins, lipids, and nucleic acids (DNA, mRNA, noncoding RNA). They are sometimes referred to as "exosomes" [58]. They mediate intercellular communication and change the molecular activity of recipient cells through the release of various biological factors [59]. Therefore, cancer cells release exosomes that contain tumor-specific biomarkers and can detect primary tumor features. Extracellular vesicles secreted by neoplastic cells can be taken up by adjacent stromal cells, leading to an alteration of the cell program [60]. 

Glioblastomas have been shown to release EVs and interact with endothelial cells to promote angiogenesis and act in an autocrine manner to stimulate tumor cell growth. Skog et al. proved that EVs can be isolated in the serum of patients with brain tumors and that specific genetic changes in the EGFR gene can be detected in EVs derived from the serum of these patients [61]. Moreover, studies in the serum of patients with glioblastoma compared to the control group showed that in the EVs of tumor patients it was possible to detect different patterns of RNA expression compared to the control group [62]. Higher plasma concentration of EVs patients with glioblastoma compared with healthy subjects has been shown and associated with tumor recurrence in patients after resection. They may be a potential biomarker to distinguish patients with glioblastoma from patients with other brain injuries and may be helpful in the early diagnosis of the disease [63].

The most commonly studied categories of extracellular vesiclesare exosomes, which range from 30 to100 nm in size, and microvesicles, which range from 100 to 1000 nm in size [64]. EVs are isolated from serum by centrifugation and purificationor precipitation. EVs can be detected using nanoparticle tracking analysis (NTA), transmission electron microscopy (TEM), and the presence of many membrane-related proteins such as ICAM-1 or integrins identified by flow cytometry or Western blot [65].

## 7. Circulating Proteins

In patients with brain tumor, the secretion of proteins may lead to an increase in the level of circulating proteins (CPs) in the blood and urine and/or CSF. Plasma-derived protein markers such as immunosuppressive acidic protein, alpha-1 acidic glycoprotein, and alpha-1 antitrypsin, the glycoprotein fibronectin, and the endothelial cell-derived thrombomodulin-1 were the first proteins to be found elevated in the blood of patients with brain tumor [66]. Then, protein markers related to angiogenesis in tumors were detected. It was found that the level of vascular endothelial growth factor (VEGF) was significantly higher in patients with brain tumor compared to healthy people, and even higher in patients with metastases in the brain [67,68]. Additionally, it has been reported that soluble VEGFR-1 (sVEGFR-1), but not sVEGFR-2, -3, and the primary fibroblast growth factor (bFGF, alternatively known as FGF-2) increased in preoperative serum samples from newly diagnosed patients with brain tumor [69]. Moreover, proteins involved in tumor cell remodeling of the extracellular matrix, such as matrix metalloproteinases (MMPs) and tissue inhibitors of metalloproteinases (TIMPs), were identified as potential diagnostic CP biomarkers. These markers help to differentiate the tumor according to its stage [70]. However, the fact that markers such as brain-derived neurotrophic factor (BDNF) and calcium-binding protein B S100 (S100B) can be detected in the circulation of healthy people negatively influences the specificity of such protein markers for detecting disease processes [71]. Potential diagnostic value has also been reported for plasma levels of interleukin 2 (IL-2) and its receptor, tumor necrosis factor alpha (TNFa), transforming growth factor beta (TGFb), chitinase-3-like protein 1 (CHI3L1, also known as YKL-40), neural cell adhesion molecule (NCAM), and neuropeptide Y (NPY) [72,73,74].

The prognostic CP markers can be divided into two subgroups: tumor-related markers and related markers with endogenous systemic stress responses. The tumor-related plasma markers YKL-40 and the extracellular domain of EGFR and osteopontin were inversely correlated with overall survival [73,75]. In addition, many CP markers implicated in tumor angiogenesis are associated with the survival of patients with glioma. The serum level of plasminogen activator inhibitor-1 (PAI-1), and the CSF protein levels of hepatocyte growth factor (HGF), were inversely associated with progression-free survival (PFS) [76]. Among these proteins, the serum YKL-40 protein appears to be the most promising prognostic marker of CP for brain tumor patients.

Circulating protein markers in patients with brain tumors can potentially also be used to monitor the effectiveness of therapy. The individual biological tumor properties and complex tissue changes induced by different cancer therapies are not adequately assessed with current MR imaging protocols. The study included patients treated with bevacizumab and irinotecan. It was found that decreased plasma levels of VEGF protein, appropriately measured, 8 weeks and 15 days after starting the treatment, were associated with an improvement in PFS and OS [77]. Unfortunately, baseline levels of VEGF were not associated with PFS in the larger AVAglio study (*n* = 571). The most extensively studied CP markers are circulating angiogenesis-related proteins. While such proteins may indeed be useful as prognostic and monitoring biomarkers for antiangiogenic therapies, a robust biomarker for the monitoring of bevacizumab treatment has not yet been reported.

## 8. Conclusions

The high incidence and mortality rate of brain tumors requires an urgent need to develop minimally invasive methods for the diagnosis and tracking of brain tumors in both primary and metastatic disease. Currently, diagnosis is based on imaging and tumor tissue data; however, there are some limitations. Conventional MRI can help surgery, but it cannot distinguish between the highly advanced tumors and can provide difficult-to-interpret results. Tumor tissue biopsies are invasive and cannot be easily repeated. Despite numerous studies on an effective biomarker for detecting and predicting brain tumors, only a few have produced promising results. High hopes in the near future are associated with the use of liquid biopsies. The liquid biopsies, such as cerebrospinal fluid and blood, can be used as potential tissue biopsy substitutes for the diagnostic and prognostic analysis of biomarkers in brain tumors. As technological advances allow for the further refinement of standardization and improved signal detection, measurement of circulating tumor biomarkers has the potential to be widely used in monitoring tumor burden. Liquid biopsies allow for multiple sampling during treatment in a noninvasive manner. In addition, a liquid biopsy may be able to reveal information about a tumor prior to clinical progression. Each of the biomarkers discussed in this review, including miRNA, ctDNA, CTC, EV, and CP, has great potential for tumor detection and prognosis. There are also other biomarkers such as neudesin, aldehyde dehydrogenase 1, and platelet-derived growth factor alpha receptor (PDGFRA), which, due to editorial restrictions, have not been described in this paper. Because each biomarker has advantages and disadvantages, acombination of markers might be beneficial. The best way seems to be to determine at least two or more markers simultaneously to increase their diagnostic utility.

Although much information has been gathered about the molecular changes in brain tumor and glioblastoma stem cells, including the availability of multiple molecular markers, caution should be taken in drawing broad sweeping conclusions regarding their clinical utility. Therefore, further research is needed into new biomarkers for the early detection of brain tumors.

## Figures and Tables

**Table 1:** Diagnostic performance of liquid biopsy in brain tumors.

Group/Markers	Significance	Study
**Molecular biomarkers**		
O-6 methylguanine-DNA methyltransferase	Prognostic and predictive biomarker	[7,8]
Epidermal growth factor receptor	Prognostic biomarker	[9,10]
Isocitrate dehydrogenase	Prognostic biomarker	[11,12]
Glial fibrillary acidic protein	Prognostic biomarker	[13,14]
Telomerase reverse transcriptase (TERT)	Prognostic biomarker	[15,16]
Loss of heterozygosity (LOH)	Prognostic and predictive biomarker	[17,18]
Tumor protein 53 (TP53)	Prognostic biomarker	[19,20]
**Circulating tumor cells**	Prognostic biomarker	[21,22]
**Circulating free DNA**	Diagnosis and monitoring response to treatment	[19,23]
**Circulating cell-free microRNAs**	Diagnosis and monitoring response to treatment	[24,25]
**Circulating extracellular vesicles**	Predictive and monitoring response to treatment	[26,27]
**Circulating proteins**	Diagnosis and monitoring response to treatment	
